# ‘Trial Exegesis’: Methods for Synthesizing Clinical and Patient Reported Outcome (PRO) Data in Trials to Inform Clinical Practice. A Systematic Review

**DOI:** 10.1371/journal.pone.0160998

**Published:** 2016-08-29

**Authors:** Angus G. K. McNair, Rhiannon C. Macefield, Natalie S. Blencowe, Sara T. Brookes, Jane M. Blazeby

**Affiliations:** 1 Centre for Surgical Research, School of Social and Community Medicine, University of Bristol, Canynge Hall, Bristol, United Kingdom; 2 Division of Surgery, Head and Neck, University Hospitals Bristol NHS Foundation Trust, Bristol, United Kingdom; Cardiff University, UNITED KINGDOM

## Abstract

**Purpose:**

The CONSORT extension for patient reported outcomes (PROs) aims to improve reporting, but guidance on the optimal integration with clinical data is lacking. This study examines in detail the reporting of PROs and clinical data from randomized controlled trials (RCTs) in gastro-intestinal cancer to inform design and reporting of combined PRO and clinical data from trials to improve the ‘take home’ message for clinicians to use in practice.

**Materials and Methods:**

The case study was undertaken in gastro-intestinal cancer trials. Well-conducted RCTs reporting PROs with validated instruments were identified and categorized into those combining PRO and clinical data in a single paper, or those separating data into linked primary and supplemental papers. Qualitative methods were developed to examine reporting of the critical interpretation of the trial results (trial exegesis) in the papers in relation of the PRO and clinical outcomes and applied to each publication category. Results were used to inform recommendations for practice.

**Results:**

From 1917 screened abstracts, 49 high quality RCTs were identified reported in 36 combined and 15 linked primary and supplemental papers. In-depth analysis of manuscript text identified three categories for understanding trial exegesis: where authors reported a “detailed”, “general”, or absent PRO rationale and integrated interpretation of clinical and PRO results. A total of 11 (30%) and 6 (16%) combined papers reported “detailed” PRO rationale and integrated interpretation of results although only 2 (14%) and 1 (7%) primary papers achieved the same standard respectively. Supplemental papers provide better information with 11 (73%) and 3 (20%) achieving “detailed” rationale and integrated interpretation of results. Supplemental papers, however, were published a median of 20 months after the primary RCT data in lower impact factor journals (median 16.8 versus 5.2).

**Conclusion:**

It is recommended that single papers, with detailed PRO rationale and integrated PRO and clinical data are published to optimize trial exegesis. Further work to examine whether this improves the use of PRO data to inform practice is needed.

## Introduction

The updated Consolidated Standards of Reporting Trials (CONSORT) extension for patient reported outcomes (PROs) aims to facilitate the use of PRO data in health policy and practice through the transparent reporting of PROs in randomized controlled trials (RCTs)[1]. It makes recommendations for reporting of PRO instrument validity, presentation and handling of missing data and reporting of PRO sample size calculations, data analyses and results within the main text and abstract. The statement endorses reporting the rationale/hypotheses for PRO assessment and it highlights the need for integrated reporting of the PROs with the clinical findings of the paper (extensions and elaborations 2a, 2b and P20/21 and 22). These latter recommendations are particularly essential for critical interpretation of the trial, so called exegesis or a “take-home message”, for clinicians to understand and use results in clinical practice.

The CONSORT extension provides illustrations of how to report these issues. For example, elaboration 2a and extension P2b state that authors should “briefly establish the rationale for including PROs and why specific outcomes were selected”, and “report the rationale for the selection of specific patient-reported outcomes”. Furthermore, the guidelines state (in items P20/21 and elaboration 22) that “the clinical significance of PRO results is often not discussed in RCT reports but should be interpreted in relation to other important clinical outcomes such as survival”. Whilst this is helpful, the level of detail required for reporting the PRO data is unclear and this may have a detrimental impact on the overall clinically relevant trial conclusions.

This problem is further compounded by the way that PRO data are published. Some trials publish results in a single paper combined with clinical findings, which may limit full explanation of PRO results in the context of finite manuscript word limits. Publication of clinical and PROs separately is therefore attractive, however, practicing oncologists may be less likely to read the supplemental paper and thus not use PRO data in decision-making. The PRO CONSORT statement does not provide guidance for PRO reporting within these different scenarios. Whether PRO data are published together with clinical outcomes or separately in two articles, there is a need for optimal reporting of clinical and PROs so that they can be used in clinical practice. The aim of this paper, therefore, is to explore current standards and make recommendations for reporting a combined PRO and clinical ‘take home’ message to use in reporting RCTs in oncology.

## Materials and Methods

This study was conducted in two parts. Part 1: systematic identification of well-designed and conducted RCTs reporting PROs with validated instruments, categorisation of papers into combined PRO and clinical reports or linked primary and supplemental reports, and assessment of PRO reporting within each RCT (PRO CONSORT extension). Part 2: development of novel methods to examine the ‘take home/trial exegesis’ message of the RCT and application to the papers identified in Part 1.

### Part 1(a) Identification of well-designed and conducted RCTs reporting PROs with validated instruments

Systematic review methodology was used to identify RCTs at a low risk of bias reporting PROs with validated instruments in radical treatments of gastro intestinal oncology. Full-text articles were obtained. Gastro-intestinal oncology trials of radical treatment were chosen because the research team were familiar with the clinical and PRO data in this area, and trials at a low risk of bias are examples of best practice.

Electronic searches were performed in MEDLINE, Embase and Cochrane databases using the OVID SP gateway and Cochrane library. Search terms for esophageal, gastric and colorectal cancer were combined, as were terms for chemotherapy, radiotherapy, surgery or combined treatment. Results were restricted with the application of terms for “randomized clinical trials” and “patient reported outcomes”, and limited to articles published between January 2000 and October 2012 (see full search in S1 Appendix). The search output was imported into Reference Manager software and duplicate records removed. References for relevant studies before the year 2000 were obtained from a previous systematic review [2]. Titles and abstracts were screened by two researchers (AGKM and RM). Serial publications for the same trial (e.g. articles reporting short and long term PROs) were included. Excluded were phase II studies, RCTs of endoscopic and non-biomedical interventions, or trials limited to palliative treatment, screening or premalignant conditions. Only English-language publications were considered. Articles were assessed for risk of bias in the trials by three researchers (AGKM, RM, NB) using the Cochrane tool [3]. Studies classified with potential high or unascertainable risk of bias were excluded. Independent data extraction was conducted by at least two reviewers (AGKM, NB, RM, JMB) using a pre-designed and piloted form. Details of the trial were recorded including disease site, treatment intervention, primary and secondary outcomes, number of participants, and main trial results. The systematic review PRISMA checklist is presented in S2 Appendix.

### (b) Categorisation of papers into combined PRO and clinical reports or linked primary and supplemental reports

Where included papers indicated the presence of previously published results from the same trial, these additional papers were sought and included in the analysis. These linked papers are hereafter considered in the order by which they were published, with those published first and second defined as “primary” and “supplemental” respectively. Thus, trials were categorized into those reporting PROs and clinical outcomes in a single, combined paper and those reporting results in linked primary and supplemental papers. The journal impact factor (Thomson Reuters, 2012) and the date of publication for each paper were recorded. Descriptive statistics compared journal impact factor for combined and linked primary and supplementary papers and median times between publications were summarized.

### (c) Assessment of PRO reporting using the new CONSORT extension

The PRO CONSORT extension was applied to all trials to establish standards of PRO reporting, with the exception of item P6a which was an inclusion criterion (use of a validated PRO measure). Reporting of item 7a (PRO sample size) was recorded as present if a sample size calculation was completed in trials with patient reported primary outcomes, or if it were not applicable (for example, if there were no PROs as primary outcomes). Descriptive statistics are presented to consider PRO CONSORT standards within trials reporting in a single, combined publication or in linked primary and supplementary papers.

### Part 2 (a) Development of methods to present the ‘take home/trial exegesis’ message of PRO with clinical data in trials

Methods to report the ‘take home’ messages of clinical and PROs in trials were developed through an in-depth analysis of items 2a and P2b (rationale/hypotheses for PRO measurement), and P20/21 and 22 (limitations and implications for clinical practice, and interpretation of PROs in relation to clinical outcomes) to identify good practice and produce methods to inform a PRO take home message from trials. All papers were read and re-read independently by at least two (AM, RM and JMB) researchers to become immersed in the data and relevant text was independently coded, copied verbatim into an electronic database and analysed for consistency between researchers. Discrepancies in coding were discussed within the study team (AM, RM and JMB). Methods for reporting trial ‘take home’ message were developed and applied iteratively to relevant text. Deviant examples were sought to challenge theories. Primary quotations are provided in accordance to methods of qualitative rigor. It was also noted whether primary papers indicated the future publication of a supplemental PRO paper (defined as “signposting”).

### (b) Application of the novel methods to included trials

The methods described above were applied to the included trials and reported data examined by whether trials were published in combined or linked primary and supplemental reports.

## Results

### Part 1(a) Identification of well-designed and conducted RCTs reporting PROs with validated instruments

**Fig 1:**
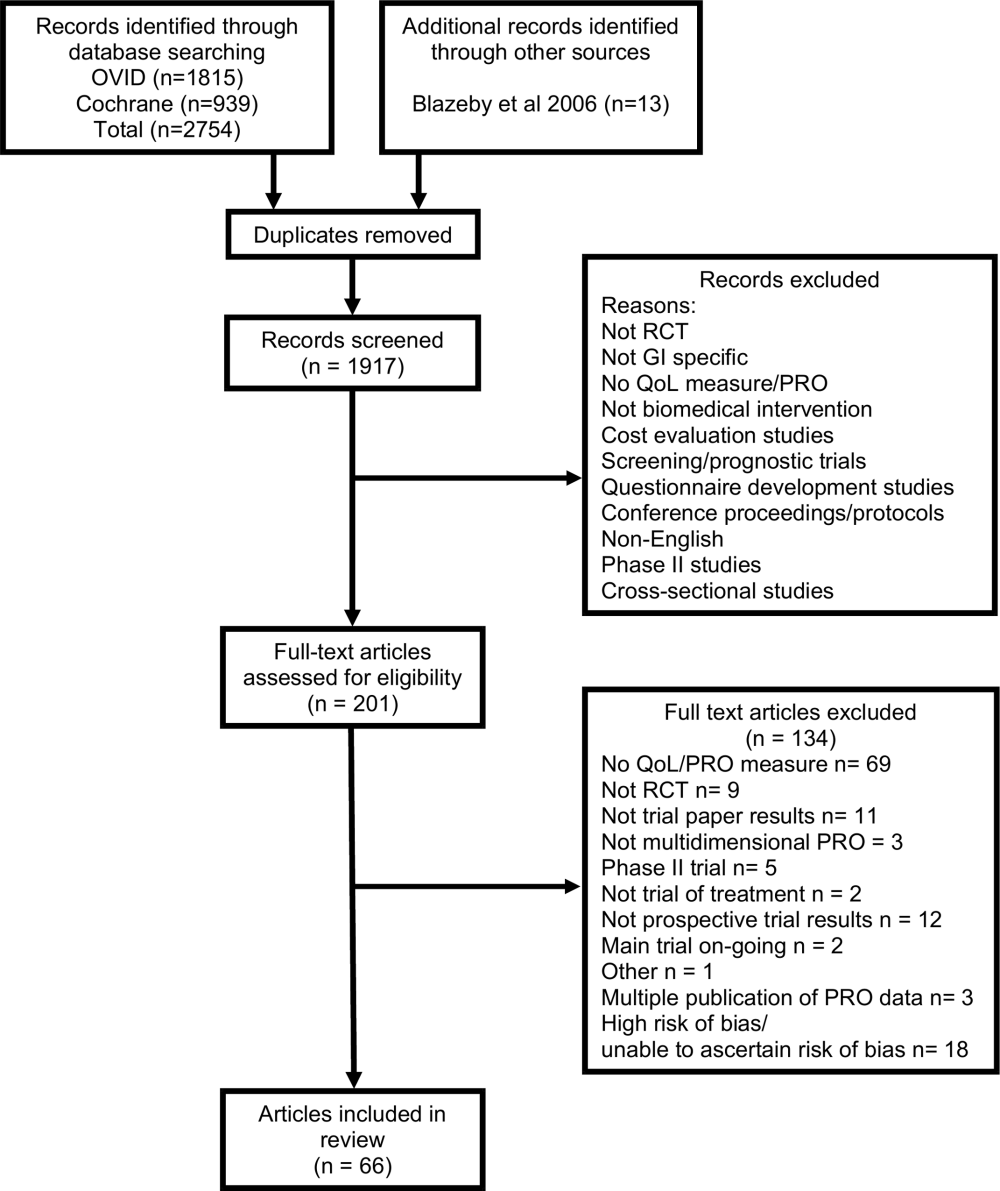
PRISMA diagram of stages of the systematic review.

OVID (MEDLINE and Embase) and Cochrane database search yields were 1815 and 939 records. After de-duplication, 1917 abstracts were screened, 1716 excluded, and 201 full text articles further assessed for eligibility, and these were supplemented with 13 studies from a previous systematic review [2]. Sixty-seven articles met the inclusion criteria describing trials at low risk of bias (Fig 1). The 66 included articles reported PROs from 49 RCTs, the majority of which were chemotherapy interventions (22/49, 44.9%) in colorectal disease (36/49, 73.5%, Table 1).

**Table 1:** Characteristics of included trials grouped by whether PRO and clinical data were presented in combined PRO and clinical paper or separate primary and supplemental papers.

	Combined clinical and PRO* paper (n = 37)	Separate clinical and PRO papers (n = 15)
	N (%)	N (%)
**Trial type**		
• Chemotherapy	14 (37)	(33)
• Radiotherapy	0	(14)
• Surgery	12 (32)	(46)
• Other^‡^	11 (30)	7 (46)
**Disease site**		
• Esophagogastric	22.4)	0
• Colorectal	27 (72)	12 (80)
**Sample size**		
• <100	8 (16)	(7)
• 100–199	4 (10)	(7)
• 200–499	13 (35)	(33)
• 500–999	8 (21)	(27)
• 1000–1999	3 (8)	(27)
• 2000–3000	1 (3)	0
**Primary outcome**		
• Survival	20 (54)	(46)
• Response rate	(8)	0
• Progression/ recurrence	6 (16)	(13)
• PRO	(16)	0
• Hospital stay	(5)	0
• 30 day post-op morbidity	0	(7)
• Diarrhea	(3)	0
• Pulmonary infection	(3)	0
• Unclear	2 (5)	0
**Journal impact factor**	Median (range)	Median (range)
• Primary paper	16.8 (2.8 to 50)	(5.1 to 50)
• Supplemental paper		5.2 (2.1 to 30)
**Time between primary and supplemental paper (months)**	n/a	20 (5 to 51)

^‡^other biochemical modulators e.g.monoclonal antibody, radioactive yttrium

* PRO: Patient reported outcome

### (b) Categorisation of papers into combined PRO and clinical reports or linked primary and supplemental reports

Some 36 (71%) single papers reported combined PRO and clinical results and the remaining 30 were linked primary and supplemental papers. The median journal impact factor for both combined and primary papers was the same (16.8), but supplemental PRO papers were reported in journals with a lower median impact factor (5.2). The mean time between publication of linked primary and supplementary papers was 20 months (range 5 to 51).

### (c) Assessment of PRO reporting using the new CONSORT extension

Overall, the reporting of most papers did not meet the new PRO CONSORT standards (S1 Table). There were six (2 combined; 0 primary and 4 supplemental) papers reporting all PRO CONSORT items (Table 2). Primary papers reported the fewest items (median 3, range 2 to 7), typically lower than combined papers (median 6, range 2 to 12) and supplemental papers (median 10, range 4 to 12). The least frequently reported items related to “results” (13a, 15, 16, 17a and 18), and were reported in less than one third of papers. The least frequently reported items related to “results” (13a, 15, 16, 17a and 18), and were reported in less than one third of papers.

**Table 2:** Analyses of reporting PRO CONSORT extension criteria, grouped by combined PRO and clinical papers, or separate primary and supplemental papers.

CONSORT PRO item		Combined clinical and PRO paper (n = 36)	Separate clinical and PRO papers (n = 15 pairs)		Total(n = 66)	
			1°	2°	
		N (%)	N (%)	N (%)	N (%)	
**P1b**	The PRO should be identified in the abstract as a primary or secondary outcome.	28 (78)	4 (27)	15 (100)	47 (71)	
2a/P2b*	The relevant background and rationale for why PROs were assessed in the RCT should be briefly described/ The PROs hypothesis should be stated and relevant domains identified, if applicable.	22 (61)	5 (33)	15 (100)	42 (64)	
P6a**	Evidence of PRO instrument validity and reliability should be provided or cited, if available.	36 (100)	15 (100)	15 (100)	66 (100)	
**7a†**	How PRO sample size was determined	33 (92)	15 (100)	15 (100)	63 (95)	
**P12a**	Statistical approaches for dealing with missing data are explicitly stated	9 (25)	1 (7)	10 (66)	20 (30)	
**13a**	The number of PRO outcome data at baseline and at subsequent time points should be made transparent	6 (17)	1 (7)	9 (60)	16 (24)	
**15**	A table showing baseline demographic and clinical characteristics for each group including PRO data	7 (19)	0	10 (67)	17 (26)	
**16**	For each group, number of participants (denominator) included in each analysis and whether the analysis was by original assigned groups	8 (22)	0	8 (53)	16 (24)	
**17a**	For multidimensional PROs, results from each domain and time point specified for analysis.	9 (25)	0	10 (67)	19 (29)	
**18**	Results of any other analyses performed, including subgroup analyses and adjusted analyses, distinguishing prespecified from exploratory including PRO analyses, where relevant	10 (28)	3 (20)	7 (46)	20 (30)	
**P20/21‡**	PRO–specific limitations and implications for generalizability and clinical practice	29 (81)	3 (20)	14 (93)	46 (70)	
**22‡**	PRO data should be interpreted in relation to clinical outcomes including survival data, where relevant	29 (81)	3 (20)	14 (93)	46 (70)	

* For more detailed analysis of items 2a/P2b see Table 3

** Study inclusion criteria

† Only applicable to trials with PROs as primary outcome (n = 6, all combined papers).

‡ For more detailed analysis of items P20/21/22 see Table 3

### Part 2 (a) Development of novel methods to examine the take home message of PRO with clinical data in trials

The new method for understanding and improving combined reporting practice was developed based upon the in-depth analyses, emergent data and iterative discussions with AM and JMB. Sections of verbatim text were coded to items 2a and P2b were classified as providing a 1) detailed rationale/hypothesis—when authors included a specific PRO domain and/or hypothesized effect, 2) general rationale/hypothesis—when authors included non-specific rationale (i.e. “to examine quality of life”) or 3) no rationale provided. Verbatim quotes of PRO rationales are presented in Tables 3 and 4. An example of a detailed rationale included: “…we hypothesized a priori that [intervention] would result in a decrease in the magnitude and rate of decline in HRQL, particularly in physical function and overall well-being”[4], and an illustration of a general rationale included “…to compare…quality of life…between [intervention] and [control]”[5].

**Table 3:** Reported PRO rationale (items 2a and P2b) and authors’ interpretation of PRO in relation to clinical findings (items P20/21 and 22) in primary reports of trials with separate primary and supplemental papers. Extracted text was abridged where appropriate, as indicated by a series of periods (…), but otherwise presented verbatim.

Author [citation]		PRO rationale	Level of detail*	Interpretation of PRO in relation to clinical findings:	Level of detail†
Ajani [6] [7]	1°	“To investigate whether adding [intervention] to [control] could improve patient outcomes (time-to-progression [TTP], overall survival [OS}, quality of life. . .” “Time to 5% definitive deterioration in global health status assed by QLQ-C30 was the primary quality of life parameter. “	Detailed	“. . . [Intervention] resulted in significantly improved TTP (primary end point), OS, and overall response rate (secondary end points), with global health status (quality of life). . . preserved for a longer time.”	Detailed
	2°	“. . .to investigate whether the better efficacy with [intervention] was counterbalanced by. . .the impact. . .on patient QOL” “The primary endpoint of the QOL assessment was. . .global health status”	Detailed	“.. significantly better preservation of QOL for patients treated with [intervention].. as a result of a significantly higher level of efficacy. . . . despite a higher incidence of some toxicities. . .”	General
Au[4] [8]	1°	“. . .no trials have demonstrated an effect of [intervention] on. . . .quality of life. . .” “The secondary end points were. . .quality of life, assessed by mean changes in scores of physical function and global health status..”	Detailed	“[Intervention] improves overall survival and progression-free survival and preserves quality of life measures. . .”	General
	2°	“…. we hypothesized a priori that [intervention] would result in a decrease in the magnitude and rate of decline in HRQL, particularly in physical function and overall well-being.”	Detailed	“Patients who received [intervention] experienced significantly less HRQL deterioration and a longer time before clinically significant deterioration occurred. These results are important, because…although [intervention]. . . results in improved OS, PFS, RR, and DCR…the magnitude of these benefits. . .was not large.” “. . .[intervention] offers clinically important survival and HRQL benefits. . .”	General
de Boer [9] [10]	1°	No PRO rationale	Absent	No integration of PRO and clinical data	Absent
	2°	“. . .to compare the quality of life of patients. . .who underwent [intervention] with patients.. who underwent [control]”	General	“..comparing. . .quality of life..is of great interest because a choice between the..two treatment options proves to be difficult..based on overall survival. However…no lasting differences in the quality of life of patients. . .were found.”	General
Braga[11] [12]	1°	“To clarify the value of [intervention]. . .quality of life. . .should be considered”	General	“[intervention] resulted in earlier postoperative recovery, better cosmesis and improved quality of life. . .compared to [control].”	General
	2°	“The primary endpoint was to compare the impact of [intervention] and [control] on 30-day postoperative morbidity.” “Recovery of social and physical activity was evaluated. . .by a specificd adaptation of the SF-36. . .”	Detailed	“. . .the [intervention] resulted in a reduction of both the overall morbidity rate and the length of hospital stay, and in a faster recovery of physical and social activity.”	Detailed
Chau [13] [14]	1°	No PRO rationale	Absent	No integration of PRO and clinical data	Absent
	2°	“. . .to assess QOL. . . .in patients receiving [intervention]”	General	“[Intervention] was associated with significantly better quality of life. . . Due to the shorter treatment duration, [intervention] had a faster time of QOL recovery. Quality adjusted survival was also in favour of the [intervention]…	General
Hallböök [15] [16]	1°	No PRO rationale	Absent	No integration of PRO and clinical data	Absent
	2°	“We hypothesized that such clear differences in clinical bowel function [with the intervention] would also be reflected in the score of a general quality of life instrument……”	Detailed	“The observed difference in clinical bowel function was not. . . reflected in an improved QOL score. . .”	General
Janson [17] [18]	1°	No PRO rationale	Absent	No integration of PRO and clinical data	Absent
	2°	“. . .with the hypothesis that [intervention] results in an improved HRQL when compared with [control]”	Detailed	“HRQL was better..after [intervention]. At present, several studies indicate that the oncologic results are at least equal after [intervention].”	General
Kabbinavar [19] [20]	1°	No PRO rationale	Absent	No integration of PRO and clinical data	Absent
	2°	“The primary HRQoL endpoint was the time to deterioration in HRQoL measured by the Colorectal Cancer Subscale score”	Detailed	“this prospective HRQoL analysis supports the clinical benefit of [intervention] in improving time to disease progression and prolonging overall survival, without compromising patients’ HRQoL”.	General
King [5] [21]	1°	“The aim of this study was to compare. . . quality of life. . .in a prospective group of patients undergoing [intervention]”	General	“Patients undergoing [intervention] stay in hospital half as long. . .with no. . .deterioration in quality of life. . .” “. . .clinical improvements resulting from [intervention] did not cause significant deterioration in quality of life. . .”	General
	2°	“. . .to compare recovery after [intervention] and [control]. . .using. . .self-report and observer data.”	General	“The earlier discharge in the [intervention] group did not result in any deterioration in quality of life outcomes compared with those in the [control] group” “Despite perioperative optimization of [control], short-term outcomes were better following [intervention]. There was no deterioration in quality of life or increased cost associated with the [intervention].”	General
Kopec [22] [23]	1°	“A secondary aim was to compare quality of life. . .”	General	No integration of PRO and clinical data	Absent
	2°	“We hypothesized that the [intervention] would be associated with higher HRQL and that it would be perceived as more convenient.” …. “The primary end point for this study was the FACT-C total score.”	Detailed	“The efficacy of the two regimens is similar, as demonstrated.. by the survival and disease-free survival analyses. . . This underscores the importance of patient-reported outcomes..” “Both regimens … do not differ in their impact on HRQL.”	General
Marijnen [24] [25]	1°	No PRO rationale	Absent	No integration of PRO and clinical data	Absent
	2°	“. . .we studies the effects of [intervention] on the HRQL and sexual functioning. . .”	Detailed	“The results of this study enable physicians and patients to weigh the beneficial effect of [intervention] on local recurrence against the price to be paid in terms of HRQL and sexual functioning.”	Detailed
Siena [26] [27]	1°	No PRO rationale	Absent	No integration of PRO and clinical data	Absent
	2°	“. . .exploratory analyses were conducted that assessed the association between [trial outcome variables] and HRQoL”	General	“. . . lack of disease progression was associated with … higher HRQoL for [intervention] patients only. . .Lack of disease progression was associated with better symptom control, HRQoL, and OS.	General
Stephens [28] [29]	1°	No PRO rationale	Absent	No integration of PRO and clinical data	Absent
	2°	“. . .the advantages of [intervention] to all patients needs to be balanced against any negative impact on patients’ quality of life”. “. . .the primary quality-of-life aims as “What is the longer-term (2-year) effect of the treatments on (1) sexual function and (2) bowel function?” Secondary outcome measures were “What is the effect of treatment on physical function and general health?” To address these questions, the sexual dysfunction and bowel function scales from the QLQ-CR38 and the physical function and general health scales from the MOS SF-36 were used.”	Detailed	“Therefore our results, together with those of the Dutch trial, provide convincing data on the impact of surgery and PRE on sexual and bowel function.” “The information presented in this article should allow clinicians to discuss with patients an estimate of the benefit of PRE in terms of reduction in LR risk balanced against the detrimental toxicity that is attributable to PRE.”	Detailed
Weeks [30] [31]	1°	No PRO rationale	Absent	“The detailed quality of life component of this trial suggests that greater benefits in terms of the quality of life and recovery may be possible if fewer procedures are converted.”	General
	2°	“The trial was also designed to test the hypothesis that [intervention] is associated with superior QOL outcomes” “the study protocol specified. . .the variability in pain distress item and the global ratings scale”	Detailed	“[Intervention].. results in statistically significant but clinically modest decreases in the duration of postoperative in-hospital analgesia and in length of stay. . . However, these differences do not translate into statistically significant improvements in symptoms or QOL. . .”	General
Wu[32] [33]	1°	No PRO rationale	Absent	No integration of PRO and clinical data	Absent
	2°	“We hypothesised that patients receiving [the intervention] would have more symptoms and greater fatigue than patients receiving [the control], with treatment arms difference most prominent at the 6-month assessment, and probably continuing up to 1 year after random assignment.”	Detailed	“Although the morbidity rate was higher in [intervention] patients than in [control] patients, our analysis indicates that [intervention] did not adversely influence QOL”	General

* Detailed rationale/hypothesis: specifying a PRO domain or hypothesized effect; general rationale/hypothesis: any other description; absent: no rationale. See methods for more details

† Interpretation was considered “detailed” where authors discussed the direction of change (e.g. increased/decreased/no change) of a specific PRO domain (e.g. physical function) in relation to the direction of change of a specific clinical outcome (e.g. survival). All other discussions, where present, were considered “partial” interpretations. Where no appropriate text was identified, interpretation was considered “absent”. See methods for more details

**Table 4:** Reported PRO rationale (items 2a and P2b) and authors’ interpretation of PRO in relation to clinical findings (items P20/21 and 22) in reports of trials with combined clinical and PRO papers. Extracted text was abridged where appropriate, as indicated by a series of periods (…), but otherwise presented verbatim.

Author [citation]	PRO rationale	Level of detail*	Interpretation of PRO in relation to clinical findings	Level of detail†
Biere [34]	“We compared [intervention] with [control]. . . to assess the rate of pulmonary infection and quality of life associated with [intervention]”	General	“In this trial, [intervention] resulted in a lower incidence of pulmonary infections 2 weeks after surgery and during stay in hospital, a shorter hospital stay, and better short-term quality of life than did [control], with no compromise in the quality of the resected specimen.” “Additionally, [intervention] preserved quality of life better than [control] did. After 6 weeks, the SF 36 questionnaire and global health experience in the EORTC C30 module were better for patients in the [intervention] group than for those in the [control] group. In the oesophageal-specific OES 18 questionnaire, pain and talking were adversely affected in patients in the [control] group as compared with those in the [intervention] group.”	Detailed
Bramhall [35]	No PRO rationale	Absent	No integration of PRO and clinical data	Absent
Carmichael [36]	No PRO rationale	Absent	“The safety advantages of [intervention] surprisingly did not lead to demonstrable improvement in quality of life.”	General
Cunningham [37]	No PRO rationale	Absent	No integration of PRO and clinical data	Absent
de Gramont [38]	“. . .to compare the two treatments in terms of. . .QoL”	General	“The [intervention] seems beneficial. . ., demonstrating a prolonged progression free survival with acceptable tolerability and maintenance of QoL.” “Median QoL scores were similar for the two arms. . ., despite the increased incidence of [treatment]-related side effects …”	General
Doeksen [39]	“The objective. . .was to compare functional and surgical results of [intervention] with [control] and their impact on quality of life.” “The primary end-point was the function. . . assessed at 12 months by the validated COlo-Rectal Functional Outcome (COREFO) questionnaire’s summary score.”	Detailed	“. . .a better functional outcome was found in patients with [intervention] than [control]. These functional differences did not influence health-related and overall quality of life.”	General
Douillard [40]	“The QLQ-C30 questionnaire was analysed with the global health status/QoL scale (QL) as the primary endpoint. . .”	Detailed	“[Intervention] was well-tolerated and increased response rate, time to progression, and survival, with a later deterioration in quality of life.” ^†^	General
Douillard [41]	No PRO rationale	Absent	“It was surprising that there was no observed difference between the treatment arms in quality of life, despite the clear reduction in toxicity with [intervention].”	General
Fein [42]	“. . .to identify optimal [treatment] in terms of quality of life”	General	“There were no differences in operative time, postoperative complications, and mortality.., there were no benefits of [intervention] in terms of quality of life, independent of the resection status. In the third, fourth, and fifth year after surgery quality of life was significantly improved for patients with [intervention].	General
Fields [43]	No PRO rationale	Absent	No integration of PRO and clinical data	Absent
Fuchs [44]	“. . .to compare. . .effect on patient quality of life of these two [treatments]”	General	“This. . . trial provides comparative data on the efficacy, tolerability, and effect on patient quality of life between the [treatments].”	General
Furst [45]	“. . .we tested [intervention] with [control] for. . . .quality of life. . .”	General	No integration of PRO and clinical data	Absent
Gray [46]	“. . .to assess whether [intervention] could. . . .change quality of life”	General	“No decrease in quality of life was observed which is in accord with the lack of serious toxicity and treatment-related complications.” “[intervention] increases treatment effectiveness when measured by tumor response and time to disease progression and suggests an increase in survival for patients surviving more than 15 months. [Intervention] does not compromise quality of life or add significant toxicity.”	General
Guillou [47]	No PRO rationale	Absent	“no differences were recorded between [control] and [intervention]. . . with respect to tumour and nodal status, short term endpoints, and quality of life.”	General
Hoksch [48]	“. . .to evaluate the quality of life during the first postoperative year comparing [intervention] and [control]”	General	“In this study of global health status and quality of life, patients operated on with [control procedure] did not reach their preoperative values compared to the patients with the [intervention]. . .” “The clinical advantage manifested 6 months after operation. . . For that reason only patients with a good long-term prognosis might benefit from [intervention].	Detailed
Jayne [49]	No PRO rationale	Absent	“[Intervention]. . . . is as effective as [control] in terms of oncological outcomes and preservation of QoL”	General
Kang [50]	No PRO rationale	Absent	“. . .[intervention] is feasible and does not increase short-term oncological risks, which are predicted by CRM positivity and macroscopic quality of TME specimens. . .The results of this trial also suggest that [intervention] results in a better quality of life for up to 3 months. . .”	General
Kataria [51]	“To compare the quality of life (QOL) in patients undergoing [intervention] with [control]. . .” “The objective of this study is to assess the QOL following [intervention]. . .”	General	No integration of PRO and clinical data	Absent
Kemeny [52]	“We hypothesized that patients in the [intervention] arm would have better physical and social functioning, fewer role limitations due to their emotional health, and better health perceptions than patients in the [control] arm.”	Detailed	“[Intervention] prolonged the median survival. . . was associated with a greater likelihood of objective tumor responses. . ., enhanced time to hepatic progression. . ., and improved physical functioning (QoL measurements).”	Detailed
Kohne [53]	No PRO rationale	Absent	No integration of PRO and clinical data	Absent
Lal [54]	“No studies have evaluated whether [intervention] is superior. . .in terms of. . .quality of life”	Detailed	“There were no improvements in failure-free survival from continuing [intervention]. . . . However,.. there was no deterioration in QoL. . .”	General
Maughan [55]	“Several specific quality-of-life endpoints were predefined in the protocol: palliation of key symptoms, toxic effects, psychological effect, functional status, social functioning, and overall quality of life”	Detailed	“[A] and [B] regimens were similar in terms of survival, quality of life, and response rates. [C] showed similar response rates and overall survival to the [A] regimen and was easier to administer, but resulted in greater toxicity and inferior quality of life.”‡ “Since there was similar overall survival, quality of life became an important outcome measure.”	General
Punt [56]	“The primary objective. . .was to examine the treatment effect on the mean global health status score. . .”	Detailed	No integration of PRO and clinical data	Absent
Punt [57]	“The primary objective. . .was to examine the treatment effect on the mean global health status score. . .”	Detailed	“[Intervention]. . .results in a small but significant improvement in progression free survival without adding toxicity or worsening QoL. . . .” ^†^	General
Rao [58]	No PRO rationale	Absent	“Although there was a trend in favor of [intervention] for progression free survival, and more patients had stable disease, this did not translate in an improved QOL or survival advantage.”	General
Ross [59]	“…we report results. . .comparing [intervention] with [control] using. . .QOL. . .as the study’s end points”	General	“The equivalent efficacy of [intervention] was demonstrated, but QOL was superior with [control].”	General
Sailer [60]	“Randomised trials. . .have shown functional superiority of [intervention]. . .it was hypothesized that significant differences in bowel function should also be reflected in quality of life” “Sample size analysis was based on. . .global health status. . .”	Detailed	“. . .patients undergoing [intervention] may not only expect better functional results but also an improved quality of life. . .”	General
Saini [61]	“. . .to assess QOL of patients undergoing [treatment]”	General	“this study has demonstrated that the [intervention] is associated with less acute toxicity and less impairment of QOL than [control]. Furthermore, this has been achieved without any obvious adverse effect on outcome”	General
Saltz [62]	No PRO rationale	Absent	“the [intervention] was associated with higher rates of tumor regression, progression-free survival, and overall survival without compromising the quality of life.”	General
Sobrero [63]	No PRO rationale	Absent	“.. Progression free survival was significantly longer in experimental. . ., while the overall survival was similar in both arms. . .; quality of life was similar as well.” ^†^	General
Sobrero [64]	No PRO rationale	Absent	“.. [intervention] reduced the risk of progression.., and improved median progression free survival. . ., and response rate.. The QOL assessments also support this benefit. Global health status as well as physical, emotional, and cognitive functioning were significantly better with [intervention].”	Detailed
Tebbutt [65]	No PRO rationale	Absent	“the addition of [intervention]. . .has no effect on response rates compared with [control]. In addition, there was no significant effect on overall survival or quality of life,. . .”	General
Tol [66]	No PRO rationale	Absent	“the [intervention] resulted in a significant decrease in progression free survival and a poorer quality of life”	General
Van Hooft [67]	“We aimed to establish whether [intervention] has better health outcomes than does [control]” “The primary outcome was mean global health status. . . assessed with the QL2 subscale of the European Organisation for Research and Treatment of Cancer quality of life questionnaire.” “This measure was chosen because the outcome of the treatments, such as need for a stoma, incisional hernia, lengthy intensive care, and hospital stay, might affect patients’ quality of life”	Detailed	“. . .[intervention] or [control] did not have any distinct benefits for global health status, mortality, morbidity, other quality of life dimensions, and stoma rates.”	Detailed
Vlug [68]	“. . .combining the [intervention] will result in the fastest postoperative recovery.”	Detailed	“Treatment groups had similar morbidity, reoperation and readmission rates, equal in-hospital mortality, comparable levels of quality of life. . .”	General
Zachariah [69]	. . . .”if [intervention] was efficacious in reducing treatment-induced diarrhea, better QoL and bowel scores were expected for the [intervention] for all instruments.”	Detailed	“We found that [intervention] did not show a statistically significant reduction in the incidence or severity of diarrhea or change in patient-reported bowel function. . .”	Detailed

* Detailed rationale/hypothesis: specifying a PRO domain or hypothesized effect; general rationale/hypothesis: any other description; absent: no rationale. See methods for more details

† Interpretation was considered “detailed” where authors discussed the direction of change (e.g. increased/decreased/no change) of a specific PRO domain (e.g. physical function) in relation to the direction of change of a specific clinical outcome (e.g. survival). All other discussions, where present, were considered “partial” interpretations. Where no appropriate text was identified, interpretation was considered “absent”. See methods for more details

Likewise, sections of text coded to the items P20/21 and 22 were developed as 1) detailed interpretations—where authors discussed the hypothesized effect of a specific PRO in relation to the hypothesized effect of a clinical outcome, 2) general interpretations–when authors include non-specific interpretation of PROs in relation to clinical outcomes, or 3) no integrated PRO and clinical interpretation of results in the paper. An example of detailed interpretation of findings includes “… [Intervention] resulted in significantly improved TTP [time to progression] (primary end point), OS [overall survival], and overall response rate (secondary end points), with global health status (quality of life)… preserved for a longer time.”[6]

### Part 2, b) Application of the methods to included trials

Of the 36 trials reporting combined papers, there were 11 (30%) papers that provided a PRO rationale/hypothesis, 10 (30%) providing general information and 15 (40%) not providing a PRO rationale (Table 5). The interpretation of PRO data in the context of clinical outcomes were detailed in six (16%), general in 24 (65%) and absent in 7 (17%) papers (Items P20/21 and 22, Table 3). There were seven papers that described both detailed PRO rationale/hypotheses and detailed interpretations of PROs in relation to clinical outcomes [5, 6, 13, 26, 30, 68, 70].

**Table 5:** Novel methods for assessing CONSORT PRO extension items 2a/P2b and P20/21/22, grouped by combined PRO and clinical papers, or linked primary and supplemental papers (n = 67).

CONSORT PRO item	Combined clinical and PRO paper (n = 36)	Linked clinical and PRO papers (n = 15 pairs)	
		1°	2°
	N (%)	N (%)	N (%)
Rationale/hypothesis (2a/P2b)*			
• Detailed	11 (31)	2 (14)	73)
• General	10 (28)	3 (20)	(27)
• Absent	15 (41)	10 (66)	0
**Interpretation of findings (P20/21/22)†**			
• Detailed	6 (17)	1 (7)	(20)
• General	2 (64)	4 (27)	80)
• Absent	7 (19)	10 (66)	0

* Detailed rationale/hypothesis: specifying a PRO domain or hypothesized effect; general rationale/hypothesis: any other description; absent: no rationale. See methods for more details

† Interpretation was considered “detailed” where authors discussed the direction of change (e.g. increased/decreased/no change) of a specific PRO domain (e.g. physical function) in relation to the direction of change of a specific clinical outcome (e.g. survival). All other discussions, where present, were considered “partial” interpretations. Where no appropriate text was identified, interpretation was considered “absent”. See methods for more details

Where PRO and clinical results were published separately (n = 15), most primary papers did not provide any PRO rationale (n = 10, 66%) or text interpreting PROs in relation to clinical findings (n = 10, 66%). One primary paper provided detailed descriptions of both these issues. In comparison, all supplemental papers described PRO rationales and most (14, 93%) contained detailed interpretation of PROs in relation to clinical findings, although only two had detailed descriptions of both of these. Of the 15 primary papers, 66% signposted the presence of the supplemental PRO report.

## Conclusions

Reporting PRO rationale linked to clinical hypotheses, and clear reporting of PRO results interpreted appropriately in the context of the clinical outcomes are critical to ensure that oncologists gather a “take-home” message to communicate to patients which encompasses clinical and PROs. This review explored this issue in detail. Patient reported outcome reporting standards were at lowest levels in primary clinical papers (where clinical trial data was reported separately to the supplementary PROs). Whilst supplemental papers provided more detail there was a 5 to 51 month delay in publication in less well cited journals, thus diminishing their impact. Most (71%) trials did report combined results, demonstrating that it is possible to do so. New methods to examine reporting of trial “take home” messages recommend that detailed information about domain specific PRO rational and interpretation with clinical data are supplied within a main trial paper to arm clinicians with relevant outcomes to use in decision-making. Authors need to be allowed space to report these details alongside clinical outcomes to inform the take home message from papers to help clinicians in practice. Where this is not appropriate for scientific reasons, for example, if primary outcome data are available before secondary PROs, then this could be explicitly stated.

Other systematic reviews have shown that PRO reporting standards are poor [71–77] which contributed to the need for the development of the PRO CONSORT extension. Similarly, other papers have recommended clear PRO hypotheses and integration with clinical findings, however, there is no empirical data presented on how to best achieve this [78]. Reviews also have examined how PROs in RCTs influence decision-making and confirm that PRO information is not used in practice [77, 79, 80]. Previous work, however, has not provided solutions to these problems or considered the conceptual reasons why PRO data are not used in practice. Theoretically, failure of PRO data to have an impact on clinical practice may stem from problems that start with the trial design and conduct, compounded by poor reporting and separate PRO and clinical publications. Further research is now needed to investigate whether improved reporting will have the desired effect of informing patient-centred care, clinical decision making and health policy decisions. For example, work could include a study directly exploring oncologists’ views of trial reports following the introduction of new reporting guidelines would be informative. Additionally in-depth research of clinical decision-making in multi-disciplinary teams or of oncology consultations may be undertaken to examine how PRO data are used.

Research needs to be targeted into each of these areas in order to understand how improvements, such as the recently published SPIRIT statement [81] for improving RCT protocols or the CONSORT PRO extension, impact clinical practice. What is clear is that cancer patients want information about PROs and indeed rate such data of similar importance to survival information [82–84]. It is therefore critical that oncologists communicate PRO data in the context of a shared doctor-patient consultation and methods to do so are being established [85–87]. This may have occurred because authors split trial results and deliberately left the PRO methodology and findings to the supplemental report.

This review included a systematic search for studies using PRISMA guidelines [88] and transparent methodology for an in-depth analyses if the textual data, but there were some limitations. Trials of radical treatments of gastrointestinal cancers were selected for analyses because the authors were familiar with the PRO and clinical data in this field. It is conceivable that including trials in other diseases or in the palliative setting may have identified different reporting standards. For example, in the palliative setting, authors may make greater reference to PROs and their integration with clinical outcomes because the main focus of treatment is not to cure disease. Further work is needed to examine this area in detail. In addition, studies with high or unascertainable risk of bias were excluded because lower standards of reporting are associated with bias and likely poor PRO reporting [89], and it is possible that important data were missed. This is considered to be unlikely however, because even the included “high-quality” trials demonstrated significant reporting weaknesses and inclusion of poor quality trials would probably not yield exemplar practice.

In summary, this review presents and evidence based way of implementing the new CONSORT PRO extension items 2a, P2b, P20/21 and 22 based on current literature. It is recommended that RCTs report domain-specific PRO rationale with anticipated treatment effects, and integrate these findings with specific clinical outcomes in a single combined report. It is acknowledged that trials are structured around their primary (often clinical) endpoints, and it is appropriate to prioritise these data at the expense of other outcomes. It seems unnecessary, however, to relegate evaluation of patient experience to reports that may be less likely to influence practice. The adoption of better standards for PRO reporting could facilitate the use of PRO data by oncologists and patients for informed decision making.

## Supporting Information

S1 Appendix OVID search strategy. (DOCX)

S2 Appendix PRISMA Checklist. (PDF)

S1 Table Reporting of CONSORT PRO extension by individual publication, including total number of appropriately reported items. (DOCX)
