# Supplementary material for: ‘Trial Exegesis’: Methods for Synthesizing Clinical and Patient Reported Outcome (PRO) Data in Trials to Inform Clinical Practice. A Systematic Review
Source: PLoS One. 2016 Aug 29;11(8):e0160998. doi: 10.1371/journal.pone.0160998 (PMC5003376; doi:10.1371/journal.pone.0160998)
Supplement: S1 Appendix — (DOCX) [file pone.0160998.s001.docx]

S1 Appendix. OVID search strategy

“Esophageal cancer”

1. exp Esophageal neoplasms/

2. (oesophag$ adj3 (cancer$ or tumo?r$ or neoplasm$ or carcinoma$ or malignan$)).tw.

3. (esophag$ adj3 (cancer$ or tumo?r$ or neoplasm$ or carcinoma$ or malignan$)).tw.

4. (oesophag$ adj3 adenocarcin$).tw.

5. (esophag$ adj3 adenocarcin$).tw.

6. or/1-5

“Gastric cancer”

1. exp Stomach Neoplasms/

2. (stomach adj3 (cancer$ or tumo?r$ or neoplasm$ or carcinoma$ or malignan$)).tw.

3. (gastric adj3 (cancer$ or tumo?r$ or neoplasm$ or carcinoma$ or malignan$)).tw.

4. (stomach adj3 adenocarcin$).tw.

5. (gastric adj3 adenocarcin$).tw.

6. or/1-5

“Colorectal cancer”

1. exp Colonic Neoplasms/

2. Colorectal Neoplasms/

3. Rectal Neoplasms/

4. ((colorect$ or colon or colonic or rect$) adj3 (cancer$ or tumo?r$ or neoplasm$ or carcinoma$ or malignan$)).tw.

5. ((colorect$ or colon or colonic or rect$) adj3 adenocarcinoma).tw.

6. or/1-5

“Chemotherapy, radiotherapy or combined therapy”

1. exp combined modality therapy/

2. exp drug therapy combination/

3. exp chemotherapy adjuvant/

4. exp radiotherapy adjuvant/

5. chemotherapy.tw.

6. radiotherapy.tw.

7. exp Radiotherapy/

8. 6 or 4 or 1 or 3 or 7 or 2 or 5

“Surgery”

1. exp Surgical Procedures, Operative/

2. resect$.tw.

3. operat$.tw.

4. surg$.tw.

5. or/1-4

“Patient reported outcomes”

1. "Quality of Life"/

2. quality of life.tw.

3. qol.tw.

4. hrql.tw.

5. hrqol.tw.

6. "Outcome Assessment (Health Care)"/

7. patient reported outcome.tw.

8. Health Status/

9. health status.tw.

10. 1 or 2 or 3 or 4 or 5 or 6 or 7 or 8 or 9

“Randomized clinical trials”

1. RANDOMIZED CONTROLLED TRIAL.pt.

2. CONTROLLED CLINICAL TRIAL.pt.

3. RANDOMIZED CONTROLLED TRIALS.sh.

4. RANDOM ALLOCATION.sh.

5. DOUBLE BLIND METHOD.sh.

6. SINGLE-BLIND METHOD.sh.

7. or/1-6

8. exp ANIMALS/ not HUMAN/

9. 7 not 8

10. CLINICAL TRIAL.pt.

11. exp CLINICAL TRIALS/

12. (clin$ adj25 trial$).ti,ab.

13. ((singl$ or doubl$ or trebl$ or tripl$) adj25 (blind$ or mask$)).ti,ab.

14. PLACEBOS.sh.

15. placebo$.ti,ab.

16. random$.ti,ab.

17. RESEARCH DESIGN.sh.

18. or/10-17

19. 18 not 8

20. 19 not 9

21. COMPARATIVE STUDY.sh.

22. exp EVALUATION STUDIES/

23. FOLLOW UP STUDIES.sh.

24. PROSPECTIVE STUDIES.sh.

25. (control$ or prospectiv$ or volunteer$).ti,ab.

26. or/21-25

27. 26 not 8

28. 27 not (9 or 20)

29. 9 or 20 or 28

Search strategy:

(“esophageal cancer” or “gastric cancer” or “colorectal cancer”) and (“Chemotherapy, radiotherapy or combined therapy” or “Surgery”) and “Patient reported outcomes” and “Randomized clinical trials”
