# Supplementary material for: ‘Trial Exegesis’: Methods for Synthesizing Clinical and Patient Reported Outcome (PRO) Data in Trials to Inform Clinical Practice. A Systematic Review
Source: PLoS One. 2016 Aug 29;11(8):e0160998. doi: 10.1371/journal.pone.0160998 (PMC5003376; doi:10.1371/journal.pone.0160998)
Supplement: S1 Table — (DOCX) [file pone.0160998.s003.docx]

S1 Table. Reporting of CONSORT PRO extension by individual publication, including total number of appropriately reported items.

|  |  | Appropriate reporting of CONSORT PRO extension number | | | | | | | | | | | |  |
| --- | --- | --- | --- | --- | --- | --- | --- | --- | --- | --- | --- | --- | --- | --- |
| First author | Paper type | qP1a | q2a/P2b | qP6a | q7a | qP12a | qP13a | q15 | q16 | q17a | q18 | P20/21 | q22 | Total |
| Fields AL[[62](#_ENREF_62)] | Combined | No | No | Yes | Yes | No | No | No | No | No | No | No | No | 2 |
| Fein M[[61](#_ENREF_61)] | Combined | Yes | Yes | Yes | No | No | No | No | Yes | No | Yes | Yes | Yes | 7 |
| Jayne DG[[68](#_ENREF_68)] | Combined | Yes | No | Yes | Yes | Yes | No | No | No | No | No | Yes | Yes | 6 |
| Guillou PJ [[66](#_ENREF_66)] | Combined | No | No | Yes | Yes | No | No | Yes | Yes | Yes | No | Yes | Yes | 7 |
| Kohne CH [[72](#_ENREF_72)] | Combined | Yes | No | Yes | Yes | No | No | No | No | No | No | No | No | 3 |
| Saini A [[80](#_ENREF_80)] | Combined | Yes | Yes | Yes | Yes | No | No | No | No | No | No | Yes | Yes | 6 |
| Fuchs CS [[63](#_ENREF_63)] | Combined | Yes | Yes | Yes | Yes | No | No | No | No | No | Yes | Yes | Yes | 7 |
| Tebbutt NC[[84](#_ENREF_84)] | Combined | Yes | No | Yes | Yes | No | No | No | No | No | No | Yes | Yes | 5 |
| Carmichael J[[55](#_ENREF_55)] | Combined | Yes | No | Yes | Yes | No | No | No | No | No | No | Yes | Yes | 5 |
| Douillard JY[[59](#_ENREF_59)] | Combined | No | Yes | Yes | Yes | Yes | No | No | No | No | Yes | Yes | Yes | 7 |
| Bramhall SR[[54](#_ENREF_54)] | Combined | No | No | Yes | Yes | No | No | No | No | No | No | No | No | 2 |
| Maughan TS[[74](#_ENREF_74)] | Combined | Yes | Yes | Yes | Yes | No | Yes | No | No | No | Yes | Yes | Yes | 8 |
| Ross P[[78](#_ENREF_78)] | Combined | Yes | Yes | Yes | Yes | No | No | No | No | No | No | Yes | Yes | 6 |
| Punt CJ[[75](#_ENREF_75)] | Combined | Yes | Yes | Yes | Yes | No | No | No | No | No | No | Yes | Yes | 6 |
| Gray B [[65](#_ENREF_65)] | Combined | Yes | Yes | Yes | Yes | No | No | No | No | No | No | Yes | Yes | 6 |
| Sobrero A[[82](#_ENREF_82)] | Combined | Yes | No | Yes | Yes | No | No | No | No | No | No | Yes | Yes | 5 |
| de Gramont[[57](#_ENREF_57)] | Combined | Yes | Yes | Yes | Yes | No | No | No | No | No | No | Yes | Yes | 6 |
| Douillard JY[[60](#_ENREF_60)] | Combined | Yes | Yes | Yes | Yes | Yes | No | No | No | No | Yes | Yes | Yes | 8 |
| Sailer M[[79](#_ENREF_79)] | Combined | Yes | Yes | Yes | Yes | No | No | Yes | Yes | Yes | Yes | Yes | Yes | 10 |
| Furst A[[64](#_ENREF_64)] | Combined | No | Yes | Yes | Yes | No | No | No | No | No | No | No | No | 3 |
| Hoksch B[[67](#_ENREF_67)] | Combined | Yes | Yes | Yes | No | No | No | No | Yes | Yes | No | Yes | Yes | 7 |
| Cunningham D[[56](#_ENREF_56)] | Combined | No | No | Yes | Yes | No | No | No | No | No | No | No | No | 2 |
| Kemeny NE[[71](#_ENREF_71)] | Combined | Yes | Yes | Yes | Yes | Yes | No | No | No | No | Yes | Yes | Yes | 8 |
| Lal R [[73](#_ENREF_73)] | Combined | Yes | Yes | Yes | Yes | No | No | Yes | Yes | Yes | No | Yes | Yes | 9 |
| Rao S [[77](#_ENREF_77)] | Combined | Yes | No | Yes | Yes | No | No | No | No | No | Yes | Yes | Yes | 6 |
| Sobrero AF[[83](#_ENREF_83)] | Combined | Yes | No | Yes | Yes | No | No | No | No | No | No | Yes | Yes | 5 |
| Tol J [[85](#_ENREF_85)] | Combined | Yes | No | Yes | Yes | No | No | No | No | No | No | Yes | Yes | 5 |
| Punt CJ [[76](#_ENREF_76)] | Combined | No | Yes | Yes | Yes | No | No | No | No | No | No | No | No | 3 |
| Saltz LB [[81](#_ENREF_81)] | Combined | Yes | No | Yes | Yes | No | Yes | No | No | No | No | Yes | Yes | 6 |
| Vlug M[[87](#_ENREF_87)] | Combined | Yes | Yes | Yes | Yes | Yes | No | No | No | No | No | Yes | Yes | 7 |
| Zachariah B[[88](#_ENREF_88)] | Combined | Yes | Yes | Yes | Yes | Yes | Yes | No | No | Yes | No | Yes | Yes | 9 |
| van Hooft J[[86](#_ENREF_86)] | Combined | Yes | Yes | Yes | Yes | Yes | Yes | Yes | Yes | Yes | Yes | Yes | Yes | 12 |
| Kataria K[[70](#_ENREF_70)] | Combined | Yes | Yes | Yes | No | No | No | Yes | Yes | Yes | No | No | No | 6 |
| Kang S[[69](#_ENREF_69)] | Combined | Yes | No | Yes | Yes | Yes | Yes | Yes | No | Yes | No | Yes | Yes | 9 |
| Doesken A[[58](#_ENREF_58)] | Combined | Yes | Yes | Yes | Yes | Yes | Yes | Yes | Yes | Yes | Yes | Yes | Yes | 12 |
| Biere S[[53](#_ENREF_53)] | Combined | No | Yes | Yes | Yes | No | No | No | No | No | No | Yes | Yes | 5 |
| Wu CW [[52](#_ENREF_52)] | Primary | No | No | Yes | Yes | No | No | No | No | No | No | No | No | 2 |
| Veldkamp [[37](#_ENREF_37)] | Primary | No | No | Yes | Yes | No | No | No | No | No | No | No | No | 2 |
| Lembersky BC[[42](#_ENREF_42)] | Primary | Yes | Yes | Yes | Yes | No | No | No | No | No | No | No | No | 4 |
| Kapiteijn E[[44](#_ENREF_44)] | Primary | No | No | Yes | Yes | No | No | No | No | No | No | No | No | 2 |
| King PM[[5](#_ENREF_5)] | Primary | Yes | Yes | Yes | Yes | Yes | No | No | No | No | No | Yes | Yes | 7 |
| Weeks JC[[49](#_ENREF_49)] | Primary | No | No | Yes | Yes | No | No | No | No | No | No | No | No | 2 |
| Hallbook O[[35](#_ENREF_35)] | Primary | No | No | Yes | Yes | No | No | No | No | No | No | No | No | 2 |
| Hulscher JBF[[29](#_ENREF_29)] | Primary | Yes | No | Yes | Yes | No | No | No | No | No | No | No | No | 3 |
| van Cutsem[[26](#_ENREF_26)] | Primary | No | Yes | Yes | Yes | No | No | No | No | No | Yes | No | No | 4 |
| Jonker DJ[[27](#_ENREF_27)] | Primary | No | Yes | Yes | Yes | No | Yes | No | No | No | Yes | Yes | Yes | 7 |
| Chau I[[33](#_ENREF_33)] | Primary | No | No | Yes | Yes | No | No | No | No | No | Yes | No | No | 3 |
| Hurwitz [[39](#_ENREF_39)] | Primary | Yes | No | Yes | Yes | No | No | No | No | No | No | No | No | 3 |
| van Cutsem[[46](#_ENREF_46)] | Primary | No | No | Yes | Yes | No | No | No | No | No | No | No | No | 2 |
| Braga M[[31](#_ENREF_31)] | Primary | No | Yes | Yes | Yes | No | No | No | No | No | No | Yes | Yes | 5 |
| Sebag-Montefiore D[[48](#_ENREF_48)] | Primary | No | No | Yes | Yes | No | No | No | No | No | No | No | No | 2 |
| Wu CW[[51](#_ENREF_51)] | Secondary | Yes | Yes | Yes | Yes | No | Yes | Yes | Yes | Yes | No | Yes | Yes | 10 |
| Janson M[[36](#_ENREF_36)] | Secondary | Yes | Yes | Yes | Yes | Yes | Yes | Yes | Yes | Yes | No | Yes | Yes | 11 |
| Kopec JA[[41](#_ENREF_41)] | Secondary | Yes | Yes | Yes | Yes | Yes | Yes | Yes | Yes | Yes | Yes | Yes | Yes | 12 |
| Marijnen CA[[43](#_ENREF_43)] | Secondary | Yes | Yes | Yes | Yes | Yes | No | Yes | No | No | No | Yes | Yes | 8 |
| King PM[[40](#_ENREF_40)] | Secondary | Yes | Yes | Yes | Yes | No | No | No | No | No | No | No | No | 4 |
| Weeks JC[[49](#_ENREF_49)] | Secondary | Yes | Yes | Yes | Yes | Yes | Yes | Yes | Yes | Yes | Yes | Yes | Yes | 12 |
| Hallbook O [[34](#_ENREF_34)] | Secondary | Yes | Yes | Yes | Yes | No | Yes | Yes | Yes | Yes | No | Yes | Yes | 10 |
| de Boer AGEM[[28](#_ENREF_28)] | Secondary | Yes | Yes | Yes | Yes | Yes | Yes | Yes | Yes | Yes | No | Yes | Yes | 11 |
| Ajani JA[[6](#_ENREF_6)] | Secondary | Yes | Yes | Yes | Yes | Yes | Yes | Yes | Yes | Yes | Yes | Yes | Yes | 12 |
| Au HJ[[4](#_ENREF_4)] | Secondary | Yes | Yes | Yes | Yes | Yes | Yes | Yes | No | Yes | Yes | Yes | Yes | 11 |
| Chau I[[33](#_ENREF_33)] | Secondary | Yes | Yes | Yes | Yes | No | No | No | No | Yes | No | Yes | Yes | 7 |
| Kabbinavar FF[[38](#_ENREF_38)] | Secondary | Yes | Yes | Yes | Yes | Yes | No | No | No | No | No | Yes | Yes | 7 |
| Siena S [[45](#_ENREF_45)] | Secondary | Yes | Yes | Yes | Yes | Yes | No | No | No | No | Yes | Yes | Yes | 8 |
| Braga M[[30](#_ENREF_30)] | Secondary | Yes | Yes | Yes | Yes | No | No | No | No | No | Yes | Yes | Yes | 7 |
| Stephens R[[47](#_ENREF_47)] | Secondary | Yes | Yes | Yes | Yes | Yes | Yes | Yes | Yes | Yes | Yes | Yes | Yes | 12 |
